# CAVES: A Novel Tool for Comparative Analysis of Variant Epitope Sequences

**DOI:** 10.3390/v14061152

**Published:** 2022-05-26

**Authors:** Katherine Li, Connor Lowey, Paul Sandstrom, Hezhao Ji

**Affiliations:** 1 National Microbiology Laboratory at JC Wilt Infectious Diseases Research Centre, Public Health Agency of Canada, Winnipeg, MB R3E 3L5, Canada; paul.sandstrom@phac-aspc.gc.ca; 2 Department of Medical Microbiology and Infectious Diseases, Max Rady College of Medicine, University of Manitoba, Winnipeg, MB R3E 0J9, Canada; 3 Independent Researcher, 208 Park West Drive, Winnipeg, MB R3Y 0T4, Canada; connor.lowey@gmail.com

**Keywords:** comparative genomics, antigenic variation, evolution, bioinformatics, computational biology, sequence analysis, epitope prediction

## Abstract

In silico methods for immune epitope prediction have become essential for vaccine and therapeutic design, but manual intra-species comparison of putative epitopes remains challenging and subject to human error. Created initially for analyzing SARS-CoV-2 variants of concern, comparative analysis of variant epitope sequences (CAVES) is a novel tool designed to carry out rapid comparative analyses of epitopes amongst closely related pathogens, substantially reducing the required time and user workload. CAVES applies a two-level analysis approach. The Level-one (L1) analysis compares two epitope prediction files, and the Level-two (L2) analysis incorporates search results from the IEDB database of experimentally confirmed epitopes. Both L1 and L2 analyses sort epitopes into categories of exact matches, partial matches, or novel epitopes based on the degree to which they match with peptides from the compared file. Furthermore, CAVES uses positional sequence data to improve its accuracy and speed, taking only a fraction of the time required by manual analyses and minimizing human error. CAVES is widely applicable for evolutionary analyses and antigenic comparisons of any closely related pathogen species. CAVES is open-source software that runs through a graphical user interface on Windows operating systems, making it widely accessible regardless of coding expertise. The CAVES source code and test dataset presented here are publicly available on the CAVES GitHub page.

## 1. Introduction

An epitope, also referred to as an antigenic determinant, is a part of an antigen recognizable by the immune system and elicits specific immune responses. In silico approaches for epitope prediction have become essential for vaccine development and the examination of antigenic evolution in pathogens of interest, especially as the Coronavirus Disease 2019 (COVID-19) pandemic progresses and the world fights to contain emerging SARS-CoV-2 variants of concern (VOCs). Exemplified by the Immune Epitope Database (IEDB), in silico epitope analysis tools are widely applied in research and development settings, attributable to their high time efficiency and cost-effectiveness in addition to their constantly improving accuracy [1].

Depending on the study in question, epitope analysis for a given protein sequence may be applied to T cell (MHC class I or II) or B cell epitope predictions [2]. When studying the relationship between two closely related pathogens, comparative analyses of their respective putative epitopes should ideally be comprehensive and look beyond a simple presence/absence approach. Several factors to consider include (1) epitope loci; (2) epitope lengths; (3) epitope sequences; (4) matching and unmatching residues; and (5) the presence of substitutions, insertions, or deletions. The number of epitopes produced by a given prediction program depends on the size of the input amino acid sequence and various parameters applied [3]. Nonetheless, the resulting list of putative epitopes for each sequence can be substantial, making it arduous and time-consuming to perform in-depth comparative analyses without computational assistance. Furthermore, attempting such comprehensive comparisons is prone to human error due to the repetitive nature of comparing long lists of epitope sequences. These challenges make detailed epitope analyses unfeasible to perform by hand, and no program currently exists to aid in these comparisons while accounting for the biological nuances of pathogen sub-variants, such as the presence of substitutions, insertions, and deletions within epitopes.

CAVES (Comparative Analysis of Variant Epitope Sequences) is an innovative tool created to automate comparative epitope analyses. Initially designed to study differences caused by mutations in the SARS-CoV-2 VOCs versus the reference strain, CAVES utilizes two levels of comparisons to organize putative epitopes into multiple informative lists showing not only to what extent the two epitope profiles match, but also if the putative epitopes have been experimentally confirmed previously. CAVES is broadly applicable for rapid comparative analyses of epitopes from any closely related pathogens or the comparison of putative epitope sequences from different prediction tools for confirmatory purposes. CAVES is publicly available at https://github.com/connor-lowey/CAVES (accessed 4 May 2022) and runs through a graphical user interface (GUI) on Windows operating systems.

**Figure 1:**
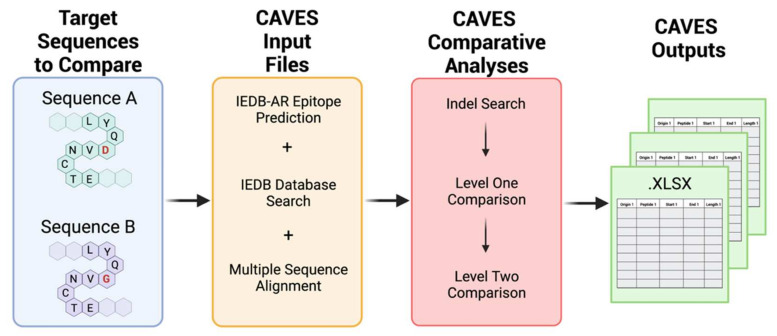
CAVES user workflow. CAVES performs comparative analyses between epitopes from two closely related sequences (sequences A and B). Target sequences are used to generate epitope predictions and database search results from the Immune Epitope Database (IEDB) resources and for multiple sequence alignment. The resulting files are input directly into the CAVES user interface for multi-level comparisons, and CAVES sorted results are output in *xlsx* format.

The CAVES workflow involves (1) the selection of target sequences to compare; (2) CAVES input file preparation; (3) CAVES comparative analyses; and (4) CAVES outputs (Figure 1). Taking a comparison of spike proteins from two SARS-CoV-2 variants as an example, here, we describe the CAVES concepts of design and workflow to address challenges of comparative epitope analysis in practice.

## 2. Materials and Methods

### 2.1. CAVES Concepts of Design

#### 2.1.1. Compatibility with the IEDB

The IEDB Analysis Resource (IEDB-AR) presents a comprehensive and user-friendly resource for epitope curation, prediction, and analysis, with most curated epitopes and references relating to infectious diseases [4,5]. The IEDB encompasses >95% of the relevant literature and spans the entire history of papers stored in PubMed [4]. The IEDB TepiTool is a step-by-step wizard for T cell epitope predictions, while the B cell epitope tool offers seven different methods, such as BepiPred-2.0 for the prediction of linear antibody epitopes [2,6].

CAVES does not conduct epitope prediction itself but instead performs in-depth comparative analysis on outputs from independent epitope prediction tools, such as those hosted by the IEDB-AR. To maximize its application potential, CAVES was designed to read output from the IEDB-AR TepiTool and the B cell linear epitope prediction tool, which encompass epitope predictions for binding to MHC class I, class II, and B cells [2,3]. The IEDB outputs are taken as the default CAVES input file format, which can easily be adapted for outputs from other similar tools (Figure 1).

#### 2.1.2. Two-Level Comparative Analysis

CAVES adopts a two-level comparison approach. In Level-one (L1), CAVES takes epitope prediction results in the form of peptide sequences directly from tools hosted by the IEDB-AR and looks for all possible matches between epitopes derived from the two target sequences being compared. In contrast, the Level-two (L2) comparison incorporates query results from the IEDB database of confirmed epitopes to determine which of the predicted epitopes used in L1 have been validated experimentally in the literature. As CAVES uses the sequence position of each epitope during comparison (Supplement 2.1), it performs an insertions/deletions (indel) check to account for any indels that would shift the epitope position numbers relative to the opposing file and adjusts these numbers accordingly during comparison.

Input Files Required for L1 Analysis: To generate input files for CAVES L1 analysis, the user must conduct T cell or B cell epitope prediction for each of the two sequences they wish to compare (hereafter referred to as sequences A and B). Epitopes should be predicted for the same protein in each sequence (the designated target protein being studied) using the same prediction tool and parameters for a fair comparison. CAVES reads the default output formats from the IEDB-AR Tepitool and B cell linear epitope prediction tool. Epitope predictions from these tools are accepted as CSV files, and CAVES will locate the columns it requires regardless of column order or the presence of additional columns not used by CAVES. CAVES uses the peptide sequence and start positions to perform comparisons, and as such, epitope predictions from other software can also be used as input so long that the user organizes a CSV file to include these columns with the same column headers, effectively mimicking the expected IEDB-AR results format (Supplement 1.1 and Figure S1).

Input Files Required for L2 Analysis: In addition to the input files for L1 analysis, CAVES L2 comparison requires two files containing query results from the IEDB database of experimentally confirmed epitopes. After CAVES performs the L1 comparison on the two epitope prediction files, it will then take the sorted lists and perform a second round of comparison using the database search results, informing the user which epitopes have experimental evidence in the published literature and which are unproven predictions. The L2 input file format is described in Supplement 1.2 and Figure S2.

Multiple Sequence Alignment (MSA): The last required file for CAVES is an MSA file consisting of sequence A, sequence B, and the database parent protein(s). CAVES uses epitope positional data to improve both its speed and accuracy. Thus, it requires the MSA file to identify any indels that would shift the amino acid positions during comparisons. While sequences A and B are the exact sequences used to generate the epitope predictions and query the database, the parent protein sequence(s) from the IEDB database must be obtained from the database search results, as described in Supplement 1.3.

Frameshift indels at the nucleotide level result in very different protein sequence(s) unsuitable for CAVES comparison. While all CAVES input data use amino acid sequences, CAVES will print a warning message in a popup box if the provided MSA file contains a large number of gap characters indicative of a poor alignment, often seen with highly dissimilar sequences (unsuitable for CAVES) or frameshifted sequences. Despite the warning, CAVES will still adjust epitope positions to account for the gap characters and perform the comparisons as usual, although the derived results of such dissimilar sequences would have limited value.

#### 2.1.3. Optional Parameter Settings

**Figure 2:**
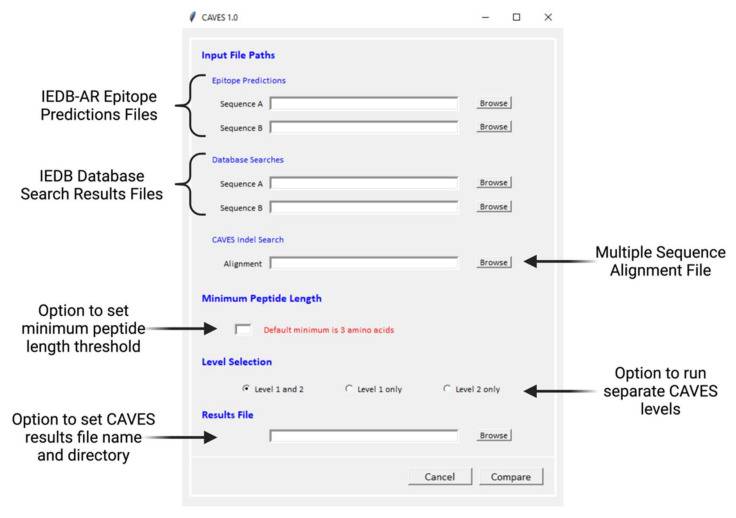
CAVES graphical user interface (GUI). CAVES opens as a single page and is compatible with Windows OS. The required fields are the epitope prediction files, database search files, and multiple sequence alignment files under the *Input File Paths* header. The *Minimum Peptide Length*, *Level Selection*, and *Results File* fields are optional parameters set to defaults unless otherwise specified.

CAVES offers several optional parameters, including a minimum peptide length threshold for CAVES analysis, the choice to run L1 only, L2 only, or both analyses, and the option to name the results file and select the target directory to save it (Figure 2, Supplement 1.4).

#### 2.1.4. CAVES Download and GUI

CAVES is open-source software written in Python v3.9 and can be run on Windows operating systems. CAVES is publicly available from GitHub (https://github.com/connor-lowey/CAVES, accessed 4 May 2022). The executable program launches as a single-page GUI with self-descriptive section titles (Figure 2). As described above, epitope prediction files, database search files, and an MSA file are required. Optional parameters, including the minimum peptide length threshold, choice of level selection, and results file name and deposition, are all available for customization. Once the “Compare” button is clicked, CAVES analysis starts, and the progress status can be viewed from the log window that opens alongside the GUI.

#### 2.1.5. CAVES Output

CAVES produces results as a multi-sheet *.xlsx* file meant to be read in Microsoft Excel (Figure S3, Supplement 1.5). This singular file contains a sheet for each of the result categories from the L1 and/or L2 comparisons, with sheets following a short-hand naming format to describe what category the sheet contains. For both L1 and L2, exact and partial match categories will display all data for a given match in a single row (Figure S3a,b). For novel category matches (L1 and L2), each novel epitope will be listed in a new row, alongside its positional data and the file from which it was derived (Figure S3c).

### 2.2. CAVES Comparative Process

#### 2.2.1. Matching Criteria

**Figure 3:**
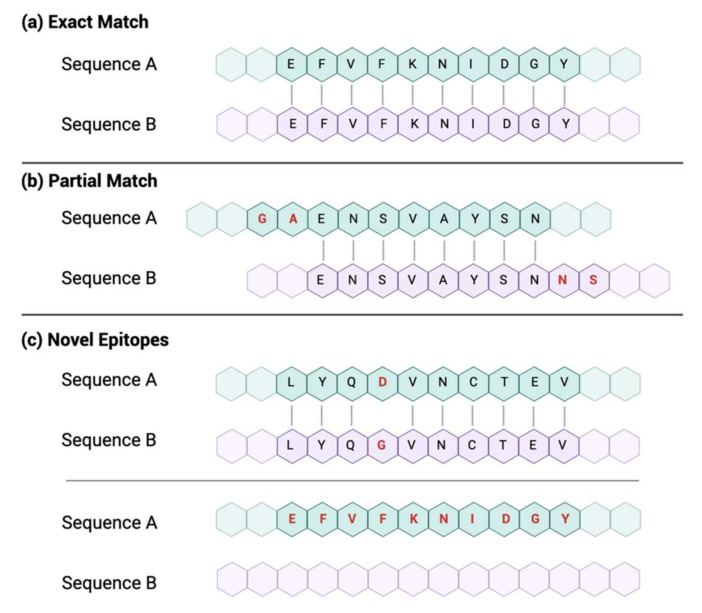
CAVES matching criteria. (**a**) An exact match occurs when two epitopes have identical amino acid characters over the entire length of at least one of the two epitopes being compared. (**b**) A partial match occurs when two epitopes have some identical amino acid characters but not enough to cover the full length of either epitope. (**c**) Novel epitopes occur either when two epitopes form an exact or partial match but contain a mutation (substitution, insertion, or deletion) in at least one position or when an epitope did not find any match in the opposing file.

To perform a comparison, CAVES takes each epitope in file A and compares it against each epitope from file B whose sequence positions are close enough to possibly contain overlapping residues. Subsequently, epitopes from both files are sorted into categories based on what matches were made. While the matching process differs slightly between L1 and L2 (described below), the three general categories that the epitopes can be sorted into are *exact match*, *partial match*, and *novel epitopes* (Figure 3, Supplement 2.2).

An *exact match* is when two epitopes have identical amino acid characters at the same loci and match for the entire length of at least one of the two compared epitopes. Conversely, *novel epitopes* could be: (A) those that did not match to anything in the opposing file or (B) those that did find a match (of any length) in the opposing file but contained a mutated amino acid, insertion, or deletion, thus making it a distinctly unique epitope. Lastly, a *partial match* occurs when two epitopes have identical characters at the same loci but are offset from each other, meaning that the match cannot cover the entire length of either epitope and the criteria for an exact match cannot be met (Figure 3).

#### 2.2.2. Level-One Comparison

**Figure 4:**
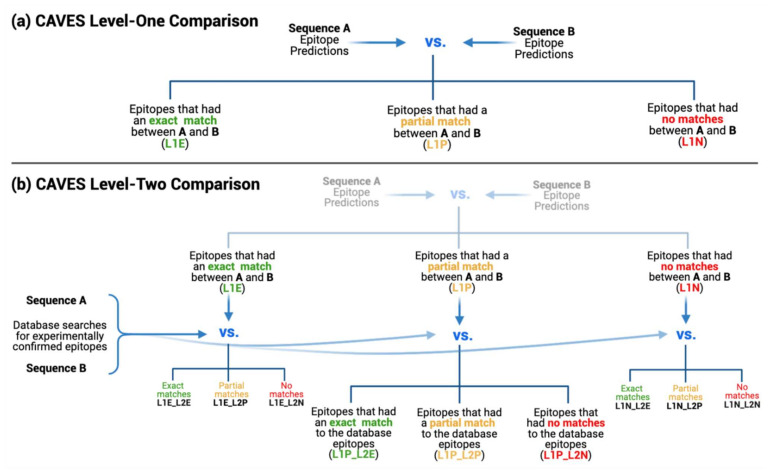
CAVES two-level comparison approach. (**a**) CAVES Level-One (L1) compares two files of epitope predictions, sorting them into categories of exact matches (L1E), partial matches (L1P), and novel epitopes (L1N). (**b**) CAVES Level-Two (L2) compares the epitopes from the three resulting L1 categories to files of database search results, sorting each L1 category into an additional triplet of exact (L2E), partial (L2P), and novel (L2N) categories.

L1 compares epitopes derived from the two prediction files, each containing a list of putative epitopes with positional information. This level takes each epitope in file A, finds all potential matches in file B, and sorts the results into three match categories. L1 is designed to be thorough and account for all match situations regardless of epitope size, overlapping amino acid count, or the presence of indels or amino acid substitutions (Figure 4a). The results from L1 comparisons make up the first three sheets in the results file, with sheet names as follows: L1E (L1 exact matches), L1P (L1 partial matches), and L1N (L1 novel epitopes). 

#### 2.2.3. Level-Two Comparison

The L2 comparison uses the three resulting categories from L1 and compares each of these to the database search files containing experimentally confirmed epitopes. L2 informs the user which epitopes from each sorted category have experimental validity and which are novel predictions (Figure 4b).

Like L1, the L2 comparison accounts for all epitope sizes, numbers of overlapping amino acids, and the presence of any mutations while sorting results into the second level of exact match (L2E), partial match (L2P), and novel results (L2N) categories. The L2 criteria for all match categories differ slightly from L1 since the L2 comparison is tailored to the sorted epitopes from L1 (L1E, L1P, or L1N) (Supplement 2.3). Accordingly, in the final *xlsx* results file, the L2 data sheets use a naming format that combines the L1 category used in the comparison, along with the L2 category produced as a result (i.e., L1E_L2E, L1E_L2P, L1E_L2N, etc.). As such, if both L1 and L2 analyses were selected, the final CAVES results file would contain 12 sheets: 3 for L1 and 9 for L2. These may be thought of in a branching pattern, where each L1 category produces its own triplet of exact, partial, and novel results in L2, as illustrated in Figure 4b.

### 2.3. Sample Dataset

#### 2.3.1. Sequence Downloads and Manipulation

Two full-length SARS-CoV-2 genomes from the National Centre for Biotechnology Information (NCBI) database, including NC_045512 (the reference sequence, Wuhan strain) and OU801816 (B.1.1.7 variant, alpha VOC), were applied for demonstration [7]. Both sequences were trimmed to the spike glycoprotein open reading frame, translated into amino acids in MEGA X, and exported as FASTA files [8].

#### 2.3.2. IEDB Epitope Prediction

Spike amino acid sequences for the two target SARS-CoV-2 strains were uploaded to IEDB-AR TepiTool (http://tools.iedb.org/tepitool/, accessed 3 October 2021) as individual runs for T cell MHC class II epitope prediction [3]. TepiTool used the NetMHCIIpan prediction method for both runs, and the predicted consensus percentile rank was set to ten [3,9,10]. Additional parameter details are available in Supplement 3.1. The TepiTool concise results (CSV format) were downloaded from both runs.

#### 2.3.3. IEDB Database Search

Separate IEDB database searches (https://www.iedb.org/, accessed 21 October 2021) were performed using the SARS-CoV-2 reference and alpha VOC spike sequences that were used for epitope prediction above [11]. For each search, the protein sequence was copied into the “*Epitope*” text box (linear peptide, substring), and parameters were set for MHC class II epitopes (as described in Supplement 3.2). Database search results were exported in CSV format.

#### 2.3.4. Multiple Sequence Alignment and CAVES Analysis

The IEDB parent sequence for the SARS-CoV-2 spike protein (in FASTA format) was obtained as described in Supplement 3.3 (NCBI accession P0DTC2). The MSA file was generated using spike sequences from the SARS-CoV-2 reference, the alpha VOC, and the IEDB parent protein with MAFFT [12]. The resulting MSA was then exported as a FASTA file. The full CAVES analysis (L1 and L2) was run as described in Supplement 3.4.

#### 2.3.5. Additional HIV-1 CAVES Analysis

An additional analysis was performed using HIV-1 sequences from the Los Alamos HIV database (https://www.hiv.lanl.gov/, accessed 25 April 2022) [13]. In brief, the envelope (Env) amino acid sequences for a subtype B reference and a subtype C reference were downloaded from the HIV database and used to perform T cell MHC class I epitope prediction with the IEDB-AR TepiTool [3,13]. For CAVES L2 input, Env region epitopes from the “Best-defined CTL/CD8 + Epitope Summary” list were downloaded from the Los Alamos HIV molecular immunology database (https://www.hiv.lanl.gov/content/immunology/, accessed 3 May 2022) [14]. The database file was formatted as described in Supplement 1.2 and used for both sequence A and B “Database Searches” fields in CAVES to determine which of the epitope predictions were already present in the Los Alamos list of best-defined epitopes. The full CAVES analysis was run with default parameters. Further analysis details are described in Supplement 3.5.

## 3. Results

### 3.1. Epitope Prediction and the IEDB Database Searches

The IEDB TepiTool produced two spike protein epitope prediction files, one for the SARS-CoV-2 reference strain and one for the alpha VOC. The reference and alpha VOC sequences were 3822 and 3813 nucleotides in length and produced 382 and 380 putative epitopes, respectively. As the panel of the 26 most frequent HLA alleles was used during both TepiTool runs, duplicate epitopes exist in each file, while the HLA allele and percentile scores differed. As such, 108 unique peptide sequences were found in both the reference and alpha VOC epitope prediction files, covering all 26 HLA alleles in the selected panel.

All epitope peptides were 15 amino acids in length (set by TepiTool) and spanned the full length of the SARS-CoV-2 spike (S) gene in both the reference and alpha VOC files. The four most frequently occurring epitopes (three predicted 10 times and one predicted 15 times) were identical in the reference and alpha VOC files, and all resided in the N-terminal domain (NTD) and receptor-binding domain (RBD) [15].

The IEDB was queried twice, producing results with 194 and 173 experimentally confirmed epitopes for the reference and alpha VOC S sequences, respectively. Database epitopes ranged from 11 to 27 amino acids in length and spanned the full length of the S gene [15].

### 3.2. CAVES L1

CAVES L1 compared epitope predictions from the reference and alpha VOC sequences, sorting all epitopes into the L1E, L1P, and L1N categories. As detailed in Table 1, L1E contained 93 exact matches, L1P contained 159 partial matches, and L1N contained 25 novel epitopes. Upon examination, all epitopes fulfilled the criteria for the L1 category they were sorted into, and no epitopes were misplaced. L1E epitopes spanned the entire S gene, with 1 in the N terminus, 50 in the S1 subunit, and 42 in the S2 subunit [15]. L1P contained 159 partial matches ranging from 1–14 amino acids of overlap, with an overlap of 8 amino acids being the most frequent length. These matches also spanned the full length of the S gene, with 1 in the N terminus, 88 in the S1 subunit, and 70 in the S2 subunit [15].

A total of 12 and 13 L1N epitopes were identified in the SARS-CoV-2 reference and alpha strains, respectively. All L1N epitopes resulted from the presence of a mutation within the alpha VOC epitope sequence as recognized by CAVES. L1N epitopes in the reference and alpha VOC strains were generally very similar peptides, usually offset by 0–3 amino acids and containing a known alpha VOC mutation. Epitopes from both strains covering eight of the nine known S mutations in the alpha VOC were sorted into L1N, as expected (Table S1) [16,17]. The remaining mutation (A570D) is associated with one putative epitope in the reference sequence and none in the alpha VOC [16]. As such, the reference epitope covering position 570 was sorted into L1P, as it partially matched with another epitope upstream (partial match length of seven amino acids). Overall, CAVES correctly identified all epitopes covering an alpha VOC mutation locus in both the reference and alpha VOC files and accurately binned these into the appropriate categories based on their respective comparisons.

In multiple occurrences, epitopes from the same originating sequence were predicted as overlapping 15-mer epitopes spanning a larger cumulative region. This was seen throughout the reference and alpha VOC epitope predictions, as these larger overlapping peptides were present in all L1 categories (L1E, L1P, and L1N). The S receptor-binding domain (RBD; position 319–541) and the receptor-binding motif (RBM; position 437–507) are of particular interest due to their interactions with the host ACE2 receptor [15,18]. The L1 results contained 18 exact matches covering the RBD region, as well as 29 partial matches (overlap ranging from 1–14 amino acids) and 2 novel epitopes. The two novel epitopes (one each from the reference and alpha VOC) were both within the RBM range of the RBD, covering the N501Y mutation [16]. Additional epitopes within the RBM included 5 exact matches and 11 partial matches.

### 3.3. CAVES L2

CAVES ran the required indel search, L1, and L2 analyses on the SARS-CoV S epitope sequences, producing results in approximately 3.6 seconds. The numbers of epitopes sorted into each L2 category are detailed in Table 1. Like L1, all epitopes fulfilled the criteria for the L2 category they were sorted into, and no epitopes were misplaced. From this, CAVES accurately identified which S epitope predictions from the reference and the alpha VOC were already present in the IEDB database query, indicating experimental confirmation in the published literature.

L1E_L2E contained L1 epitopes that were exact matches between the reference and the alpha VOC, additionally finding an exact match to a pre-existing database epitope in L2. This category contained 82 putative epitopes (42 from the reference and 40 from the alpha VOC), spanning both the S1 and S2 subunits of the S gene [15]. Thirty-six epitopes had sequences overlapping the RBD, with eight of these within the RBM [15,18].

L1E_L2P contained epitopes that were exact matches in L1 but found only partial matches with database epitopes in L2. Consequently, this category was large, with 444 partial matches spanning the full length of the S gene [15]. These were often overlapping epitope predictions that found multiple partial matches with a single database epitope, as discussed in Supplement 2.3. Partial matches ranged from 1–14 amino acids in length, with 12 as the most common length.

L1E_L2N contained 15 L1E epitopes that did not have any degree of match with the database epitopes in L2 (Table S2). This included seven epitopes from the reference and eight from the alpha VOC, mostly located on the terminal ends of the S gene [15]. Eleven were in the S1 subunit, with none found in the RBD but two spanning the Furin cleavage site [15]. This includes an alpha VOC epitope directly after the D614G mutation (617–631 with respect to the reference sequence) that did not find any matches with L2 database epitopes, but the corresponding reference strain epitope from L1E had exact and partial matches in L2 [17]. Additionally, four epitopes were in the S2 subunit, all of which were within the last 151 residues of the gene [15].

L1P_L2E reported 85 putative epitopes from L1P that had exact matches to database epitopes in L2 (44 from the reference and 41 from the alpha VOC). Of these, 58 were within the S1 subunit, with 39 in the RBD and 11 of these covering the RBM [15,18]. The remaining 27 matches were spaced throughout the S2 subunit.

L1P_L2P contained 462 epitopes from L1P that had partial matches to database epitopes in L2 (229 from the reference and 233 from the alpha VOC). Match lengths spanned from 1–14 amino acids, with 12 as the most common match length and matches covering the full length of the S gene [15].

L1P_L1N contained partially matching epitopes from L1 that did not find a match with any database epitope in L2. This included 16 epitopes, 7 from the reference and 9 from the alpha VOC, with 13 located in the S1 subunit and 3 in the S2 subunit. L1P_L2N included the reference sequence epitope covering the A570D region described in the L1 results above [16]. Additionally, two epitopes bridged the Furin cleavage site into the S2 subunit [15].

L1N_L1E contained unique putative epitopes from L1N that had exact matches to database epitopes in L2. This included six matches, all of which involved L1N epitopes derived from the reference sequence. Three matches were within the S1 subunit, and three were in the S2 subunit [15]. These epitopes covered regions of the del69–70, del144, S982A, and D1118H alpha VOC mutations, although no alpha VOC epitopes were present in L1N_L1E [16].

L1N_L2P reported 49 L1N epitopes (28 from the reference and 21 from the alpha VOC) that found partial matches to database epitopes in L2. Match lengths spanned from 1–14 amino acids with overlaps of 1, 6, 9, 10, and 14 tied for the most common length. Thirty-one matches were within the S1 subunit, with eight of these in the RBM and five covering the Furin cleavage site [15,18]. Eighteen additional matches were in the S2 subunit [15]. These epitopes covered regions of the del69–70, del144, N501Y, D614G, P681H, T716I, S82A, and D1118H alpha VOC mutations [16,17].

L1N_L2N contained three epitopes from L1N that were unable to match to any database epitopes in L2. These epitopes were all from the alpha VOC and covered the del69–70, D614G, and S982A mutations, as described in Table S2 [16,17]. Two were located within the S1 subunit, with the remaining epitope in the S2 subunit [15].

### 3.4. Additional HIV-1 CAVES Analysis Results

Env epitope prediction with the IEDB-AR Tepitool produced 679 epitopes for subtype B and 653 epitopes for subtype C. As the panel of the 27 most frequent A and B alleles was used during both TepiTool runs, duplicate epitopes existed in each file with different HLA alleles and percentile scores. Without duplicates, the subtype B and C files contained 242 and 229 unique epitopes, respectively. The Los Alamos database list of best-described CTL/CD8 + epitopes contained 42 epitopes within the Env region that were used in CAVES L2 analysis.

CAVES ran the required indel search, L1, and L2 analyses on the HIV-1 Env epitope sequences, producing results in approximately 5.8 seconds. All epitopes fulfilled the criteria for the L1 and L2 categories they were sorted into, and no epitopes were misplaced. All mismatched positions were successfully reported by CAVES to be in the L1N category and corresponded to mutations previously identified (by manual analysis) between the subtype B and C sequences. The number of epitopes sorted into each CAVES results category is detailed in Table S3.

## 4. Discussion

Pairwise comparison of highly similar sequences from pathogen sub-variants, directly based on their predicted epitope profiles, allows for analysis of their antigenic evolution, primarily driven by host immunity. By comparing putative epitopes of the spike proteins from two SARS-CoV-2 lineages, we present CAVES as a novel tool and demonstrate its usage to address the challenges in comparative epitope analysis of two closely related viral variants. This work is further supported by an additional comparative analysis of putative epitopes from the envelope proteins of two HIV-1 subtype references. CAVES fills a gap in the current public toolset as, to the best of our knowledge, no other program currently exists to compare variant epitope sequences while accommodating substitutions, insertions, and deletions that alter the amino acid sequence. We showed that CAVES L1 analysis successfully compared all putative epitopes in both the SARS-CoV-2 and HIV-1 analyses and accurately binned them into the exact match (L1E), partial match (L1P), or novel (L1N) categories. Furthermore, CAVES L2 comparison using the database search results will quickly distinguish, for all L1 epitope match categories, which epitopes have or have not been experimentally validated, effectively allowing users to identify novel areas of study and accelerate global research efforts. CAVES allows users to run only one of its two levels of comparison or both as a bundle to satisfy varied application needs.

From an evolutionary perspective, these data are very informative in defining the evolutionary trend of SARS-CoV-2. For instance, the protein regions where the L1E and L1P epitopes reside could be considered relatively conservative. In contrast, the L1N epitopes present only in the earlier reference strain were lost when the virus evolved into the alpha lineage. Conversely, the L1N epitopes found only in the alpha VOC were gained when the virus mutated. This hypothesis is well-supported by the observation that all signature mutations in the alpha strain are associated with the novel epitopes identified by CAVES [16,17]. It is logical to consider that those L2N epitopes that have not been validated previously, regardless of the L1 match categories that they belong to, should be of significance for further investigation.

While initially designed to study SARS-CoV-2 VOCs, CAVES will undoubtedly be applicable to any emerging or re-emerging SARS-CoV-2 variants, which require continued monitoring of epitope changes to better understand how the virus evolves antigenically as the pandemic continues. Apart from SARS-CoV-2, CAVES is also broadly applicable to any other viral or bacterial pathogens with sub-lineages or variants of similar genomic/proteomic sequences, as demonstrated here by the additional analysis of HIV-1 epitopes. Likewise, the CAVES methods presented here in the context of SARS-CoV-2 evolution could be applied for evolutionary analysis of any similar pathogens so long as the compared sequences are similar enough to allow for meaningful comparison. Moreover, CAVES simplifies the workflow of such comparative analyses and significantly improves time efficiency, making in silico methods much more feasible for a broader range of studies.

The IEDB-AR was used for its exemplary epitope prediction tools, and the resulting output formats were defined as the standard input for CAVES. Notably, CAVES could be easily adapted to compare the output from any epitope prediction tools for in-depth comparison or computational validation. There are many computational tools available for T cell epitope prediction, and the IEDB-AR B cell linear epitope prediction tool itself hosts seven different methods to choose from [19]. Oftentimes, studies will predict epitopes for the same sequence using several different in silico methods and consider those that are commonly predicted by multiple programs with higher confidence, especially when working with B cell epitope prediction tools, whose performance tends to be rather poor in comparison to their T cell counterparts [4,20,21,22]. In such instances, users simply need to display their epitope predictions with the required headers in CSV format (Figure S1) and run CAVES as an L1-only comparison.

It is important to note that the sequence positions for any IEDB database-derived epitopes used in CAVES L2 are not relative to the given input sequence used to query the IEDB but instead to the source protein that the epitope was cataloged under in the database. The IEDB utilizes NCBI taxonomy and accessions to provide standardized nomenclature for protein data wherever possible and provides these with each epitope record [5]. Using the provided MSA file, CAVES aligns epitope positions amongst sequence A, sequence B, and the database-derived epitopes, allowing sequences with in-frame indels to be accurately compared. Nonetheless, the user should be mindful when reading sequence positions in CAVES L2 results.

CAVES identifies unique and matching epitopes, given input data derived from epitope prediction tools and IEDB epitope database inquiry. Consequently, the ability of CAVES to find biologically relevant matches is limited by the epitope prediction data it is provided with, and unsuitable input data will not yield meaningful results. For instance, the quality and application value of CAVES L1 results would depend on the epitope prediction tool used for CAVES input data generation. Similarly, the accuracy and breadth of the L2 results would be determined by the comprehensiveness of the IEDB database of confirmed epitopes at any given time. As such, the results demonstrated here will only be reproducible while the IEDB-AR epitope prediction tools and the IEDB database entries remain unchanged or when pre-made files from the provided sample dataset are used.

It is acknowledged that both CAVES L1 and L2 comparisons render a large amount of data in the partial match categories, which can be tedious to sort through. With epitopes in these categories, we prioritized the comprehensiveness of CAVES over the simplicity of its results and sorted all matches that did not fulfill the exact match or novel criteria into this middle category. While it is justifiable to focus only on the exact match and novel epitopes with CAVES, we believe that partial matches may still contain meaningful information. For example, if comparing two epitopes that both have a length of 15 amino acids, it is plausible that a match of 14 or 13 amino acids may still be useful for the study purpose. In contrast, a match of only one or two amino acids may be negligible for the user. Rather than setting a length threshold for defining what constitutes a partial match, we leave it to the user to decide how much overlap is required to be relevant. As CAVES results are produced in Excel spreadsheets, we strongly suggest taking advantage of the Excel filter function to aid in this analysis.

CAVES was designed for the comparative analysis of closely related but genetically distinct sequences, and thus, any mutations, including in-frame indels, contained within putative epitopes could be of significance for studying antigenic evolution in mutated regions of the genome. By reading the start positions of each epitope and knowing where any indels occur, CAVES will reposition the sequence locations accordingly to accommodate shifted sequences during the matching process. Using sequence positions also allows CAVES to speed up its search process by only comparing epitopes from overlapping regions. While CAVES accounts for in-frame indels, it will not accommodate frameshift nucleotide mutations that lead to fundamentally new, unrelated proteins that are thus unsuitable for CAVES comparison. Recognizably, this inaugural version of CAVES only accommodates the analysis of datasets derived from two original protein sequences for each run, which limits its application if working with large numbers of sequences.

## 5. Conclusions

In conclusion, here, we present a novel bioinformatics tool that automates the comparative analysis of putative epitope profiles derived from relevant sequences with high similarity. While initially designed to compare SARS-CoV-2 variants, CAVES is applicable for antigenic evolutionary analyses of any pathogens with sub-lineages or sub-variants. This freely available software represents a new addition to the public toolbox that addresses the increasing need for in-depth immune epitope analysis.

## Figures and Tables

**Table 1:** Number of epitopes in each category as determined by CAVES comparison.

CAVES Results Category ^a^	Number of Epitopes ^b^	Number Derived from Reference Sequence	Number Derived from Alpha VOC Sequence
L1E	93		
L1P	159		
L1N	25	12	13
L1E_L2E	82	42	40
L1E_L2P	444	229	215
L1E_L2N	15	7	8
L1P_L2E	85	44	41
L1P_L2P	462	229	233
L1P_L2N	16	7	9
L1N_L2E	6	6	0
L1N_L2P	49	28	21
L1N_L2N	3	0	3

**^a^** CAVES results categories naming as follows: L1/2, L1, or L2; E, exact matches; P, partial matches; N, novel epitopes. ^b^ Refers to pairs of matching epitopes for all exact and partial match categories, including duplicate epitopes that found multiple matches. Refers to individual unique epitopes for novel categories.

## Data Availability

The full sample dataset and CAVES results presented in this study are openly available on the CAVES GitHub https://github.com/connor-lowey/CAVES.

## Supplementary Materials

The supplementary materials can be downloaded at https://www.mdpi.com/article/10.3390/v14061152/s1, Figure S1: CAVES epitope prediction format; Figure S2: CAVES database search results input file format; Figure S3: CAVES output file format; Table S1: CAVES sorted results for putative epitopes covering mutations in the SARS-CoV-2 alpha VOC spike gene sequence; Table S2: CAVES L1E_L2N and L1N_L2N epitopes with associated gene regions and mutation loci; Table S3: Number of epitopes in each category as determined by CAVES HIV-1 comparison.
