# Supplementary material for: CAVES: A Novel Tool for Comparative Analysis of Variant Epitope Sequences
Source: Viruses. 2022 May 26;14(6):1152. doi: 10.3390/v14061152 (PMC9227564; doi:10.3390/v14061152)
Supplement: Supplementary file 1 [file viruses-14-01152-s001.zip › viruses-1637395-supplementary.pdf]

1. CAVES Concepts of Design

1.1. L1 Input Files

To run both the L1 and L2 analyses, CAVES requires a total of 5 input files: two epitope prediction files, two files of database search results, and one MSA file. For L1, CAVES accepts the default results format from the IEDB-AR TepiTool (any of the concise, complete, and non-redundant download options in CSV format) and B cell linear epitope prediction tool. Notably, the IEDB-AR B cell linear epitope prediction tool does not have a downloadable CSV file of predicted peptides available. In this case, users may copy from the IEDB-AR results page, paste the predicted peptide table directly into any spreadsheet software, and save it as a CSV file. If using epitope prediction software from outside the IEDB-AR, CAVES input files should be formatted to mimic the IEDB-AR format, as described in Suppl. Figure S1.

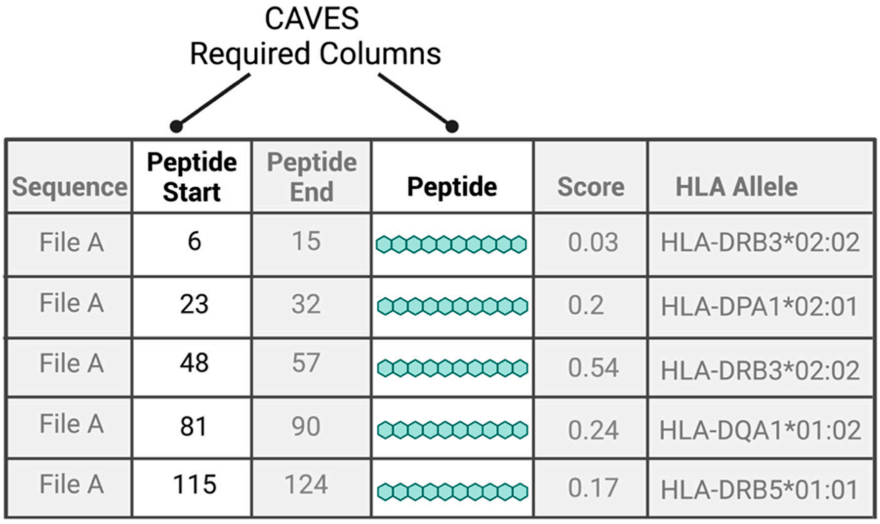

Suppl. Figure S1. CAVES epitope predictions input file format. CAVES is compatible with the IEDB-AR TepiTool and B cell linear epitope prediction tool, and is designed to read their default naming conventions. If using other epitope prediction tools, epitope predictions must be arranged as shown in CSV file format. CAVES requires the *peptide start* and *peptide sequence* columns to run but ignores all other columns (such as *peptide end*, *score*, etc.) and does not require any specific column order. Column headers (case sensitive) accepted by CAVES for the epitope sequence column include “Peptide”, “peptide”, and “Peptide sequence”; column headers accepted by CAVES for the epitope start positions column include “Peptide start”, “Start”, and “start”.

1.2. L2 Input Files

For L2, the exact sequence A and B used to generate epitope predictions should be used for the IEDB database queries, generating two unique results files. The amino acid sequence should be pasted into the Linear peptide box, and the Substring option should be selected from the dropdown menu. Search parameters (such as T cell or B cell assay,

MHC class I or II, etc.) should reflect the type of epitopes previously predicted to allow for appropriate comparisons. Search results can be downloaded from the IEDB as a CSV file and uploaded directly into the appropriate fields in CAVES GUI (Figure S2). Like L1, search results from other databases outside the IEDB or a list pre-determined by the user can be used with CAVES so long as the file is arranged appropriately. Database search results files must be in CSV format with column headers printed in row two (the IEDB format includes two header rows). The expected column headers are "Description" for the epitope sequence column, and "Starting Position" for the epitope start positions column (Suppl. Figure S2)

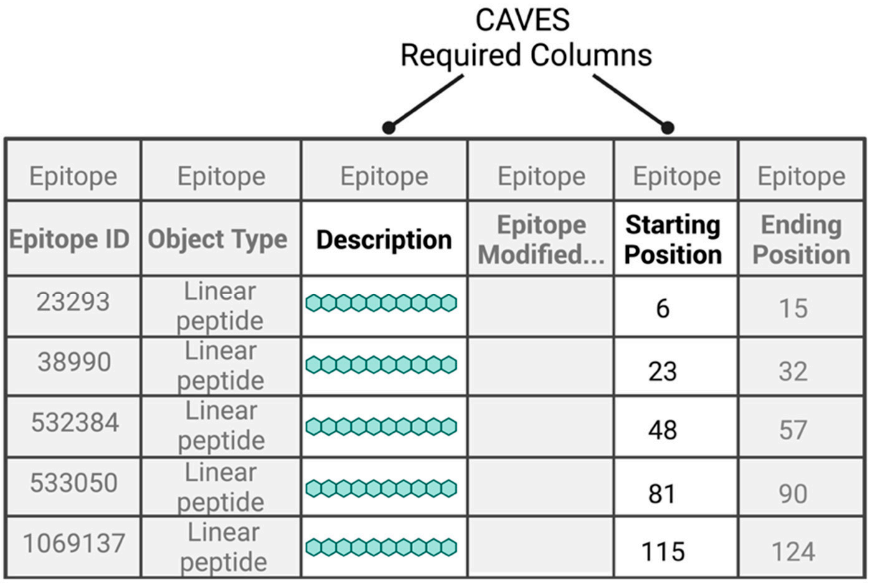

Suppl. Figure S2. CAVES database search results input file format. CAVES is compatible with the IEDB database of experimentally confirmed epitopes and is designed to read its default naming convention. If using other epitope databases or a precompiled list, epitopes must be arranged as shown in CSV file format. CAVES requires the *Description* and *Starting Position* columns to run but ignores all other columns (such as *Epitope ID, Ending Position*, etc.) and does not require any specific column order. CAVES accepts "Description" for the epitope sequence column header, and "Starting Position" for the epitope start positions column header. Headers must be placed in the second row to mimic the IEDB results format, as shown.

1.3. Multiple Sequence Alignment (MSA) File

CAVES requires a MSA file in order to account for any insertions or deletions that would shift epitope sequence positions used during the CAVES comparative process. This MSA file must contain the sequence A and B used to generate the L1 epitope predictions and the L2 database search results, as well as the database parent protein(s) from the IEDB database search results. Within each downloaded search results CSV file, users can navigate to the Parent Protein Accession column and copy the provided NCBI accession. The accession can be searched on the NCBI website

(https://www.ncbi.nlm.nih.gov/) to find the corresponding database entry. Here, the parent protein sequence can be obtained in FASTA format, which can be used to generate the MSA required by CAVES

If sequence A and B list different parent proteins, both parent protein sequences should be included in the MSA. If the two database searches list the same parent protein, the amino acid sequence only needs to be included once. The alignment should be constructed using amino acid sequences with the order of appearance as sequence A, sequence B, and lastly the database parent protein(s) (for sequence A, then B if using multiple parent proteins), as CAVES uses this sequence order to apply insertion and deletion positions to the correct epitopes during comparison. This can be done with any sequence alignment program (such as MAFFT), so long as the program uses the standard dash (-) gap character, organizes the output in the same sequence order as the input, and provides the MSA as a FASTA file (https://mafft.cbrc.jp/alignment/software/).

1.4. Optional Parameters

CAVES optional parameters include a minimum peptide length threshold, a choice of which CAVES levels to run, and the option to name the results file and choose a target directory. The minimum peptide length threshold expects a numeric value indicating the minimum length of amino acids that should be included in CAVES comparisons. Any epitopes that fall below this threshold will be ignored by CAVES and not included in the sorted results. For T cell epitope predictions, the IEDB-AR TepiTool sets the length of epitopes to be predicted as part of its own parameters, so a CAVES threshold may not be needed. On the other hand, the IEDB-AR B cell linear epitope prediction tool does not let users choose the length of predictions, and any residues with a high enough score can be included in the output regardless of length. In this case, setting a minimum length threshold with CAVES will allow users to easily filter out shorter peptides from their sorted results. If this field is left blank, CAVES sets the default threshold to three, meaning that only epitopes three amino acids or longer will be included in the results.

The level selection parameter gives the choice to run only L1 or L2 rather than the full CAVES comparison. If users want to compare two files of epitope predictions but are not interested in a comparison with database files, or alternatively, want to compare a single prediction file to a database search, but do not want to compare against another set of predictions, CAVES allows users to run only L1 or L2. As such, unnecessary fields will be removed from the Input File Paths section so that only required fields remain. Consequently, the required MSA file for the indel search only needs to include the sequences being used for the respective comparison. For example, the Level 1 only option will need an alignment file containing sequence A and B, whereas the Level 2 only option will need a file containing sequence A and the database parent protein. By default, CAVES will run both L1 and L2, and expects all input file fields to be used.

Lastly, users have the option to name their CAVES results file and choose the directory in which to save it with the browse button. If left blank, CAVES will assign a default name and write the results file in the same directory as the CAVES executable.

1.5. CAVES Output

CAVES results are written as a multi-sheet .xlsx file where each sheet contains a results category from the L1 and/or L2 analyses. It is important to note that in the case of duplicate epitope predictions (i.e., the same epitope sequence with the same positional data, listed multiple times in an epitope prediction file), CAVES will only print the duplicate sequence once in the results to avoid redundancy. This situation can occur when working with T cell epitope predictions that included multiple HLA alleles during the predictive process, thus allowing the identical epitope sequence at the same loci to be repeated for each unique HLA allele it corresponds to. CAVES output format for the exact, partial, and novel categories are displayed in Suppl. Figure S3.

2. CAVES Comparative Process

2.1. Sequence Location Data

CAVES uses epitope sequence positions to improve both its speed and accuracy during comparisons. While longer epitope sequences are less likely to find matches in distant gene regions by chance, peptides that are only a few amino acids long have a greater risk of accidentally matching to unrelated regions, effectively producing false positive matches. Reading sequence positions allows CAVES to ensure that the epitopes being compared are from the same gene loci, thus improving the accuracy of the sorted results. Furthermore, CAVES reduces its run time by only comparing sequence regions that are close enough to possibly contain overlapping residues in the opposing file, rather than searching every epitope throughout the full gene length. In order to correctly read sequence positions, CAVES performs an indel search using the provided MSA file. This allows CAVES to locate any insertions or deletions that occur and adjust the epitope positions accordingly.

2.2. Matching Criteria

CAVES comparative process sorts epitopes into categories of exact matches, partial matches, or novel epitopes based on the degree to which an epitope matches to peptides in the opposing file. An exact match is when two epitopes have the identical amino acid characters at the same loci and match for the entire length of at least one of the two epitopes. This allows CAVES to accommodate epitopes predicted at any length, regardless of whether the two epitopes being compared have even or uneven lengths.

Novel epitopes are labelled when either an epitope was not able to find any match in the opposing file, or when two epitopes did match but contained at least one mutation (substitution, insertion, or deletion), thus making the two epitopes distinctly unique. Mutations within predicted epitopes can be highly important for studying how epitope predictions change or stay the same between variant sequences, and CAVES was designed to easily find these mutated regions. As such, CAVES sorts epitopes containing mutations into the novel category even if the other amino acid residues were normal matches.

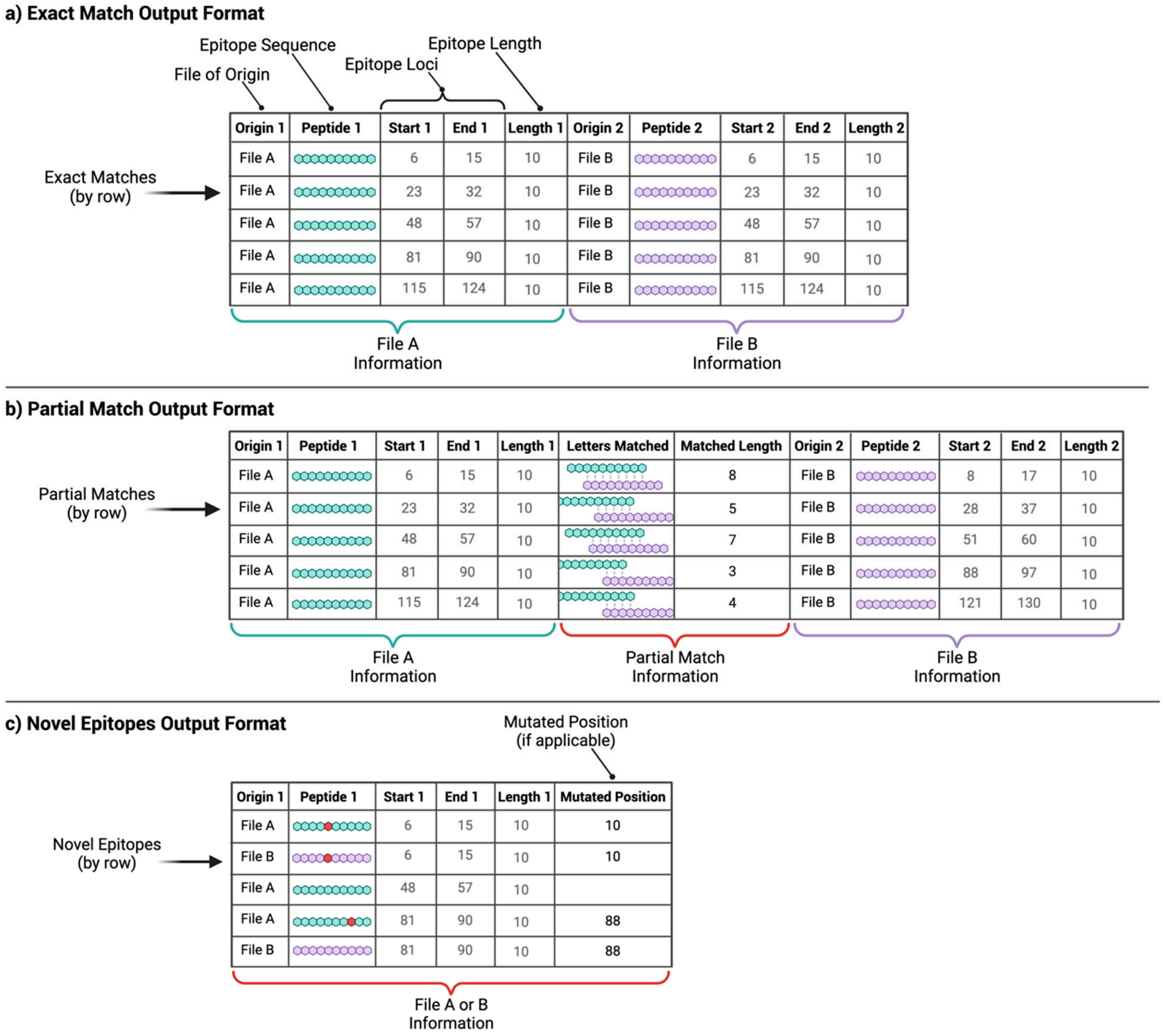

Suppl. Figure S3. CAVES output file format. **(a)** and **(b)** Exact and partial match categories display each match by row with all corresponding data for both matching epitopes. Partial matches contain two additional columns with details specific to each match. **(c)** Novel epitopes are listed as a single epitope per row, alongside its respective data and the mutated position that was mismatched (only in L1) if applicable. The mutated position field will be left blank if epitopes were labelled as novel due to no match (rather than a mutation).

A partial match is when two epitopes are offset and have some identical amino acid residues that match, but not enough to cover the full length of either of the two epitopes. This ranges from epitopes that match on only one position, to those that match on all but one position of the full length. While including this partial match category does require more direct effort from the user to decide which epitopes are true matches or novel predictions, it allows CAVES to be more applicable and accurate for a broader audience. As CAVES results are produced in Excel spreadsheets, the Excel filter function can be used to filter matches by the Matched Length column which may aid in this analysis.

2.3. Comparison Details

As mentioned previously, an exact match requires an epitope sequence to match the opposing epitope on every character of its entire length. In L1, this complete coverage can occur in either of the two epitopes being compared (those from file A or file B) whereas in L2, the epitope being fully covered must be derived from the sorted lists of putative epitopes generated in L1, not those from the database file. This approach stems from the different objectives of the two levels. Where L1 compares two similar files of epitope predictions, L2 aims to determine which of those predictions are present in the database and as such, results concerning the degree to which database epitopes are covered by various predictions are not inherently useful for CAVES objectives.

If an epitope in L1 is found to have multiple matches, all matches will be included in the final output and thus a given epitope may be found multiple times amongst multiple output categories. Conversely, in L2, if an epitope from the sorted L1 lists finds an exact match to an epitope from the database search file, any subsequent partial matches will not be recorded. In L2, an exact match is the highest priority as it provides the closest example of a similar epitope with experimental confirmation in published literature, whereas any additional partial matches are less informative and no longer needed. If no exact matches are found, partial matches and novel epitopes will still be determined as previously described, allowing for the inclusion of multiple partial matches where applicable.

This was demonstrated by the sample dataset (Table S1), where each triplet of L2 results (ex. L1E_L2E, L1E_L2P, and L1E_L2N) collectively contained more epitopes than the single L1 category they were derived from (ex. L1E). Epitopes from each L1 category found multiple matches to database epitopes in the L2 comparison, particularly in the partial match categories. This can occur when epitopes are predicted as overlapping peptides in a short range of sequence, consequently creating multiple partial matches to epitopes in the opposing file. Furthermore, matching epitopes from L1E and L1P are individually compared to database epitopes in L2, creating larger numbers of matches in L2 categories.

3. Sample Dataset Methodology

The full sample dataset and CAVES results presented in this study are openly available on the CAVES GitHub https://github.com/connor-lowey/CAVES.

3.1. Epitope Prediction Parameters

The following parameters were applied in the IEDB-AR TepiTool for both the reference strain and alpha VOC spike sequences. Host species: human; Allele class: class II; Alleles: pre-selected panel of alleles, panel of 26 most frequent alleles; Peptides: default settings for low number of peptides (15mers only, duplicates removed); Prediction method: IEDB recommended; Selection of predicted peptides: based on predicted consensus percentile rank, cutoff = 10.

3.2. Database Search Parameters

The following parameters were applied in the IEDB database searches for experimentally confirmed epitopes. Assay: T Cell, MHC Ligand, positive outcome; Epitope Source: SARS-CoV-2 organism, spike glycoprotein antigen; MHC restriction: class II; Host: human; Disease: infectious.

3.3. Multiple Sequence Alignment Parameters

The parent protein accession was obtained from the appropriate column in the reference sequence database search results. Accession P0DTC2 was searched on the NCBI website to find the corresponding GenPept entry. The FASTA sequence was copied into a text file along with the SARS-CoV-2 reference and alpha VOC spike sequences used for the previous epitope predictions and database searches (ordered as reference, alpha VOC, parent protein). The alpha VOC database search results listed the same database parent protein, so the parent protein sequence was only included once. The text file was saved as a FASTA file and uploaded as input to the MAFFT alignment webserver. The MAFFT output order was set to "Same as input", and all other parameters were left as default. The resulting MSA file was downloaded as a FASTA file.

3.4. CAVES Parameters

Under "Input File Paths", the epitope predictions and database search files were uploaded to their respective fields such that the SARS-CoV-2 reference files were uploaded as "Sequence A", and the alpha VOC files were uploaded as "Sequence B" (all in CSV format). Under "Insertions and Deletions", the MAFFT MSA file was uploaded to the "Alignment file" field. The minimum peptide length threshold was left blank (default of three amino acids), and the levels selection was left as "Level 1 and 2".

3.5. Additional HIV-1 CAVES Analysis

Two HIV-1 sequences were used in an additional CAVES analysis to support its suitability for various pathogens. Subtype B (accession AY173951) and subtype C (accession AY772699) Env amino acid reference sequences were downloaded from the Los Alamos HIV database. Each sequence was uploaded to the IEDB-AR TepiTool as individual runs with the following parameters: Host species: human; Allele class: class I; Alleles: panel of 27 most frequent A and B alleles; Peptides: default settings for low number of peptides (9mers only, duplicates removed); Prediction method: IEDB recommended; Selection of predicted peptides: based on predicted consensus percentile rank, cutoff = 1. Each concise results file was downloaded in CSV format.

Database epitopes were obtained from the Los Alamos HIV molecular immunology database using the table of best-defined CTL/CD8+ epitopes. The table was downloaded and shortened to only contain epitopes within the Env sequence region. The file was then formatted as described in Supplement 1.2 by adding a filler row and changing the names of the column headers as necessary. The MSA file was generated with MAFFT using the subtype B and C Env sequences, as well as the HIV-1 HXB2 Env sequence obtained from the Los Alamos HIV database (accession K03455) as the database parent protein. The MAFFT output order was set to "Same as input", and all other parameters were left as default. The resulting MSA file was downloaded as a FASTA file.

Files were uploaded to CAVES such that the subtype B epitope predictions were set as "Sequence A" and the subtype C epitope predictions were set as "Sequence B". The same database file was uploaded to both database fields in order to identify epitope predictions that were already present in the Los Alamos list of best-defined epitopes, regardless of subtype. The MAFFT MSA file was uploaded to the "Alignment file" field, and all other parameters were left as default.

4. Sample Dataset Results

Suppl. Table S1. CAVES sorted results for putative epitopes covering mutations in the SARS-CoV-2 alpha VOC spike gene sequence.

Type	Mutation[a]	CAVES L1 results category	Number of epitopes covering locus (reference sequence)	Number of epitopes covering locus (alpha VOC)
Deletion	69-70	L1N	2	2
Deletion	144	L1N	2	2
Substitution	N501Y	L1N	1	1
Substitution	A570D	L1P	1	0
Substitution	D614G	L1N	1	1
Substitution	P681H	L1N	1	1
Substitution	T716I	L1N	2	3
Substitution	S982A	L1N	2	2
Substitution	D1118H	L1N	1	1

[a] Known alpha VOC mutations reported by cov-lineages.org and the US Centers for Disease Control and Prevention (O'Toole *et al.*, 2021; Galloway *et al.*, 2021).

Suppl. Table S2. CAVES L1E_L2N and L1N_L2N epitopes with associated gene regions and mutation loci.

Epitope sequence	CAVES results category	Sequence of origin	Loci[a]	Spike gene region	Alpha VOC mutation[b]
DSSSGWTAGAAAYYV	L1E_L2N	Alpha VOC	250 - 264	S1, NTD	
	L1E_L2N	Reference	253 - 267		
AYYVGYLQPRTFLLK	L1E_L2N	Alpha VOC	261 - 275		
	L1E_L2N	Reference	264 - 278		
LKYNENGTITDAVDC	L1E_L2N	Alpha VOC	274 - 288		
	L1E_L2N	Reference	277 - 291		
TITDAVDCALDPLSE	L1E_L2N	Alpha VOC	281 - 295		
	L1E_L2N	Reference	284 - 298		
CTEVPVAIHADQLTP	L1E_L2N	Alpha VOC	614 - 628	S1, SD2	
ARSVASQSIIAYTMS	L1E_L2N	Alpha VOC	681 - 695	S1 – S2, Furin cleavage site	
	L1E_L2N	Reference	684 - 698		
AGLIAIVMVTIMLCC	L1E_L2N	Alpha VOC	1219 - 1233	S2, TM	
	L1E_L2N	Reference	1222 - 1236		
DDSEPVLKGVKLHYT	L1E_L2N	Alpha VOC	1256 - 1270	S2, Cytoplasm domain	
	L1E_L2N	Reference	1259 - 1273		
VTWFHAISGTNGTKR	L1N_L2N	Alpha VOC	62 - 76	S1, NTD	del 69-70
GTNTSNQVAVLYQG	L1N_L2N	Alpha VOC	598 - 612	S1, SD2	D614G
ARLDKVEAEVQIDRL	L1N_L2N	Alpha VOC	979 - 993	S2, HR1	S982A

[a] Loci with respect to the originating sequence. Alpha VOC positions will be offset from reference sequence positions after deletions at 69-70 and 144. [b] Mutation loci written with respect to reference sequence gene positions, as reported by cov-lineages.org and the US Centers for Disease Control and Prevention (O'Toole *et al.*, 2021; Galloway *et al.*, 2021).

Spike gene abbreviations: S1, S1 subunit; NTD, N-terminal domain; SD2, Subdomain 2; S2, S2 subunit; TM, transmembrane domain; HR1, heptapeptide repeat sequence 1 (Huang *et al.*, 2020; Lokman *et al.*, 2020).

Suppl. Table S3. Number of epitopes in each category as determined by CAVES HIV-1 comparison.

CAVES results category [a]	Number of epitopes [b]	Number derived from the subtype B sequence	Number derived from the subtype C sequence
**L1E**	27		
**L1P**	141		
**L1N**	417	215	202
**L1E_L2E**	8	4	4
**L1E_L2P**	34	17	17
**L1E_L2N**	32	16	16
**L1P_L2E**	9	4	5
**L1P_L2P**	44	17	27
**L1P_L2N**	75	16	59
**L1N_L2E**	19	16	3
**L1N_L2P**	106	66	40
**L1N_L2N**	329	156	173

[a] CAVES results categories naming as follows: L1/2, L1 or L2; E, exact matches; P, partial matches; N, novel epitopes. [b] Refers to pairs of matching epitopes for all exact and partial match categories, including duplicate epitopes that found multiple matches. Refers to individual unique epitopes for novel categories.
